# Notes and News

**Published:** 1902-07-26

**Authors:** 


### NOTES AND NEWS.

THE Metropolitan Hospital Sunday Fund now amounts to £58,400, including Mr. George Herring's donation of £11,000.

AT the annual distribution of prizes and conversazione at the Royal Dental Hospital of London, held last week, Sir Arthur Rucker distributed the awards and delivered an interesting address.

ON Saturday, the 19th inst., the Bishop of Kensington unveiled a window erected by Lady Measom in the Chapel of the Cancer Hospital to the memory of her late husband Sir George S. Measom. The memorial is a single light stained glass window representing a well-designed figure of Faith, and is the work of Messrs. Clayton and Bell.

IN May and June last, an International Conference of the Red Cross Societies was held at St. Petersburg. A resolution was adopted urging that no Government should permit the granting of patents or trade marks bearing the insignia and name of the Red Cross, and that those already in use should be cancelled. The measure desired is intended to prevent the abuse of the emblem of the Red Cross.

THE outbreak of cholera near Assiout is serious news. The epidemic was really established by July 15th, since when upwards of 100 cases have been reported, with a heavy mortality. The Mecca pilgrims are held responsible in Egypt, whilst a reported case of the same disease at Naples is said to emanate from China. The plague has very few victims now in Egypt, the few cases that there are being mostly in Alexandria.

EMPLOYERS of labour have a great opportunity of assisting in the prevention of epidemics of small-pox. It would be a simple matter to make vaccination within a reasonable period a condition of service. They would indirectly benefit themselves, as the heavy expenses incurred in dealing with small-pox by hospital treatment would gradually disappear from the rôle of the ratepayer. Some large employers are awaking to their duty in this respect and at the Devonport Dockyard, for instance, men employed must submit to re-vaccination.

A PHARMACOLOGICAL SOCIETY has been founded in London, for the study of the chemical and therapeutical properties of plants and chemical agents. It will meet at the premises of the Society of Apothecaries in Blackfriars. Sir W. Thiselton Dyer will be President.

A NICE little story comes from New Orleans to the effect that some of the muleteers engaged in the export of mules, horses, and cattle for the use of the British during the recent war in South Africa were employed by Boer agents to inoculate the animals with the virus of anthrax and glanders, with the interesting result, however, of rendering the restocking of the Boer farms a matter of greater difficulty than might otherwise have been the case.

THE Earl and Countess of Jersey entertained last week, in the grounds of Osterley Park, six hundred boys and girls and the staff from the various homes and ships of the National Refuges for Homeless and Destitute Children, of which Society Lord Jersey is president. A special feature of the proceedings was the presentation by Lady Jersey of prizes given by Her Royal Highness the Princess of Wales to those boys and girls who had best acted up to the motto of the Mayflower Guild "Be good and do good."

THE Natural Study Exhibition Association are holding an interesting conference in the Royal Botanic Gardens from July 24th to August 5th. The chair will be taken each day at 3 P.M. by various distinguished persons, amongst whom are Lords Strathcona and Reay. The selected speakers are all conversant with the subjects before the conference which is mainly devoted to furthering the study of nature amongst the young. "School Gardens" by Mr. T. Rooper, M.H.I. should be an interesting topic from a health aspect, as it is becoming understood that gardening in schools can be made both a healthful and profitable recreation for children. The Exhibition is under the patronage of the Queen, and the Duke and Duchess of Devonshire assist in the ceremonies.

KING EDWARD'S HOSPITAL FUND is, we learn, to become the recipient of £100 through an amusing journalistic error. It is, unfortunately, sometimes the case that criticisms forestall events, especially in monthly journals. Quite recently the *Lady's Realm* published a full and unfavourable account of the Coronation Opera performance, a performance which never took place. Messrs. Hutchinson, the proprietors of the journal have paid £100 to the Grand Opera Syndicate by way of making substantial the apology they have made in the daily papers to the syndicate. All's well that ends well, and no harm will have been done, as it happens, whilst the King's Fund will be the gainer. The writer of such criticism cannot justly be punished by proprietors of such journals, at any rate, as they must know perfectly well when demanding up-to-date accounts of current events, that the copy has to be in the hands of the printer so long before the appearance of the journal as to make it impossible to describe current events without having recourse to the imagination.

